# Mapping Advertising Assets Project: a cross-sectional analysis of food-related outdoor advertising and the relationship with deprivation in Leeds, UK

**DOI:** 10.1017/S1368980025100670

**Published:** 2025-09-03

**Authors:** Victoria L. Jenneson, Imani Wilson, Francesca L. Pontin, Charlotte E.L. Evans, Michelle A. Morris

**Affiliations:** 1 School of Food Science and Nutrition, University of Leeds, Woodhouse Lane, Leeds LS2 9JT, UK; 2 Leeds Institute for Data Analytics, University of Leeds, Woodhouse Lane, Leeds LS2 9JT, UK; 3 School of Geography, University of Leeds, Woodhouse Lane, Leeds LS2 9JT, UK; 4 Purple Nutrition, 17 Hazel Drive, Chesterfield S40 3EN, UK

**Keywords:** Advertising, Food advertising, Diet behaviour, Marketing, Policy, Food environment

## Abstract

**Objective:**

Food environments can influence dietary behaviours. Promotion of foods high in fats, salt and sugars is a barrier to healthy eating. We explore advertising by deprivation in an English city.

**Design:**

Using a cross-sectional design, we describe the prevalence of outdoor advertising, the types of products advertised and the UK Nutrient Profile Modelling scores for advertised foods and non-alcoholic beverages. Differences in outdoor advertising prevalence by area deprivation were assessed using *χ* ^2^ tests.

**Setting:**

Six areas in each of five deprivation strata were randomly selected from all 482 Leeds neighbourhoods (England) (*n* 30 neighbourhoods).

**Participants:**

Eligible outdoor advertisement assets (intentionally placed permanent/semi-permanent advertisements visible from the street) were photographed in May–June 2023.

**Results:**

A total of 295 outdoor advertising assets were recorded. The most deprived quintile had the highest number of advertising assets (*n* 74). Bus shelters were the most prevalent asset (*n* 68). The number of food adverts differed significantly by deprivation level. The two most deprived areas had higher than expected exposure, while the two least deprived areas had lower than expected exposure (*P* < 0·01). Data were insufficient to compare compliance against a hypothetical Healthier Food Advertising Policy; however, bus shelters were most likely to display high in fats, salt and sugars food adverts.

**Conclusions:**

Food advertising in Leeds is unequally distributed, with more food adverts in more deprived areas. Similar inequalities may exist in other cities, but data are scarce. Unhealthy adverts are most prevalent on bus shelters, highlighting an important asset for policy focus.

Childhood obesity is one of the greatest health concerns in the UK^(1)^, with children from the most deprived households twice as likely to be living with obesity compared with the least deprived^(1)^. For many people in the UK, the ‘physical, economic, policy and sociocultural surroundings’ that make up their local food environment^(2)^ do not reflect government healthy eating guidelines. Less healthy foods and drinks are more affordable, more readily available and more frequently marketed than healthier options^(3)^, contributing to obesity risk^(3)^. The food environment can therefore become a barrier to eating a healthy balanced diet, particularly for people in more deprived communities who likely live in the most obesogenic environments^(4)^.

In 2017 alone, £143 million was spent on advertising foods and drinks high in fats, salt and sugars (HFSS) by the leading eighteen UK brands, thirty times the spend on healthier food advertising^(5)^. HFSS food marketing is associated with increased rates of obesity via subconscious^(6)^ changes in food attitudes and preferences^(7)^, purchasing behaviours^(8)^ and consumption^(9)^.

Children are particularly vulnerable to the effects of food marketing due to their inability to comprehend marketing strategies^(9)^. Effects on food consumption in children can be immediate^(8)^, and while small, may contribute cumulatively to increased daily energy intake over time, leading to obesity. To date, UK policies restricting food advertising have specifically focused on protecting children. For example, the UK Food Standards Agency’s 2004–2005 Nutrient Profile Model (NPM) forms the basis of Ofcom’s restrictions on food advertising during children’s television programmes^(10)^. However, children are not only exposed to advertising in child-centred settings^(11)^, and food marketing exposure is a problem across society, not just in childhood. While evidence in adults is less robust^(9)^, an association appears to exist between food advertising and long-term behavioural changes in adolescents^(12)^ and adults^(13)^, including increased snacking and displacement to non-nutritious foods^(14)^. This suggests there is an unmet need for advertising strategies that promote health across all age groups.

In 2020, the government’s obesity strategy announced further legislation on HFSS advertising. New policy actions include recent retail product placement legislation^(15)^, a planned strengthening of existing television advertising restrictions and a ban on volume-based price promotions (e.g. ‘buy one get one free’)^(15)^. While promising, outdoor advertising has been largely ignored at the national level^(16)^, risking the displacement of advertising to unregulated outdoor advertising spaces.

Outdoor (out-of-home) advertising has no universal definition in the literature, but in most instances, there is overlap where they generally refer to paid-for advertisements found on assets like billboards, posters, digital screens, public transport and bus shelters, some of which may be government or local authority owned^(17)^. Differences tend to relate to adverts inside venues with an entry fee or ticket requirement, such as sports stadiums or train station platforms or moving adverts, such as those on the sides of buses. Outdoor advertising is particularly concerning due to the inability of the general public to avoid it as they go about their daily lives^(17)^. An estimated 98 % of the UK population is exposed to outdoor advertisements each week^(18)^. Furthermore, international evidence suggests outdoor food advertising targets children, with high prevalence around schools^(19)^ and other areas frequently visited by young people^(20)^. Evidence also suggests outdoor food advertising is more prevalent in deprived areas and those with high ethnic minority populations, potentially contributing to inequalities in diet and obesity^(4)^.

While there is no mandatory restriction, voluntary UK guidance for outdoor food advertising does exist. The Advertising Standards Agency’s Committee of Advertising Practice advises that outdoor advertising of HFSS products should be restricted in areas where more than 25 % of the audience is likely to include children (< 16 years of age)^(21)^. However, guidance has been criticised for ignoring other areas where children gather, such as sports arenas, city centres, parks and nurseries^(22)^. Furthermore, with responsibility falling to local authorities, adherence to UK outdoor food advertising guidance is largely unmonitored^(23)^.

Evidence around outdoor food advertising exposure in the UK is also lacking^(23)^ and differs by local context. For example, in Scotland, studies found no relationship between school location and food/drink advertisements on bus stops, nor evidence for socio-economic patterning of the advert locations. However, those from more deprived areas were subject to greater exposure to these less healthy advertisements through greater public transport usage^(24,25)^.

In February 2019, with guidance from Sustain, London introduced restrictions on advertising HFSS foods (and relevant brand-only advertising) across the Transport for London (Tf L) network. This included the Tf L Rail network, London Underground, TfL-run vehicles (buses and some taxis) and outdoor spaces (e.g. bus shelters)^(26)^. Following the Tf L ‘junk food ban’, seven London Boroughs (Greenwich, Haringey, Southwark, Merton, Luton, Tower Hamlets and Newham), Bristol (2021) and Barnsley (2022) have since introduced their own Healthier Food Advertising Policies (HFAP) on council-owned or leased sites, with varying restrictions on what may be advertised.

With HFAP being relatively new and few, evidence of effectiveness is in its infancy. Baseline data have been gathered for the city of Bristol^(27)^, while the TfL policy has been evaluated more extensively^(28)^. Results point to success across a range of indicators. Using data from Kantar’s Fast Moving Consumer Goods Panel, evidence from London shows a significant decrease in the proportion of households purchasing HFSS products (or significant attenuation of the rate in growth of HFSS purchasing) relative to pre-policy trends and control areas with no restrictions^(28)^. Reductions equate to 80·7 g of sugar and 1001 kcal removed from weekly household shops, with the greatest decline seen for chocolate and confectionery at 19·4 %^(28)^.

HFAP could provide a valuable opportunity to protect the most vulnerable in our population from over-consumption of unhealthy products and their associated health harms. But with local evidence on current outdoor food advertising exposures limited, more research is needed in a UK context.

This study investigates the outdoor food advertising environment in the city of Leeds in the North of England, which does not have an HFAP. Leeds is ethnically diverse and is ranked the 33rd (out of 317) most deprived local authority nationally^(29)^. Beginning with the hypothesis that overall advertising and food advertising prevalence are highest in areas where people experience the highest levels of disadvantage, the study aimed to:

Collect data on outdoor advertising assets across Leeds, their locations and advert features.

Investigate the area demographic determinants of total advertising and food advertising exposure.

## Methods

### Primary data collection

Following a cross-sectional observational design, this study involved photographing outdoor advertising assets in selected areas across Leeds during a 5-week fieldwork period in May–June 2023. Data collection followed an *a priori* protocol^(30)^ informed by the Best-ReMaP protocol^(31)^.

#### Area sampling strategy

As it was not possible to visit every area of Leeds, we employed a two-step stratified sampling approach to select 30 Leeds neighbourhoods, specifically Lower Layer Super Output Areas (LSOA), representing up to 3000 people, or 1200 households^(32)^. We ensured coverage of diverse areas based on their geographic location and socio-economic factors using the Index of Multiple Deprivation (IMD)^(29)^, a ranking of relative deprivation across England (see online supplementary material, Supplemental Table 1 for full list).

Leeds LSOAs were stratified into five IMD quintiles (Q1–5), where one is the most deprived. Six LSOA were selected from each IMD quintile for data collection, ensuring only one per ward (a larger geographical unit which LSOA sit within) to maximise geographic coverage across the city. For IMD Qs 2–5, six LSOA per quintile were selected at random, while for Q1, the most deprived LSOA from each of Leeds City Council’s six Priority Wards (Leeds City Council internal documentation) were selected, representing those among the 1 % most deprived nationally (listed in online supplementary material, Supplemental Table 2)^(33)^.

#### Data collection fieldwork

Data collection was conducted on foot by four members of the research team (IW, VJ, MM and CE) who were assigned LSOA based on availability and geographic access. Walking routes within LSOA boundaries were determined by a geographical information software (QGIS version 3.22.8)^(34)^ with an average walking speed (3 mph) to estimate the required data collection time. The May–June period was selected to avoid UK national holidays (e.g. Easter and Christmas), which are associated with high advertising for HFSS seasonal treats. Previous research has shown that advertising promotions typically take place in 2-week cycles^(35)^, during which areas may be saturated by large advertising campaigns, meaning little variety is observed. Conducting data collection over 5 weeks represents approximately two advertising cycles, offering a better representation of exposures without introducing seasonal variance.

To reduce bias, the order in which the thirty selected LSOA were visited was determined via block randomisation (see online supplementary material, Supplemental Table 3). A 10 % sample, three of thirty LSOA were randomly selected as a validation sub-sample. These were visited independently by two members of the research team and captured adverts compared with assessing the reliability of the data collection method.

#### Data collection pilot

A pilot phase was conducted in two LSOA (one small urban and one large rural) prior to data collection to assess the accuracy of walk time estimates, informing the data collection timeline and allowing issues to be identified. Following the pilot, the data collection timeframe was extended to accommodate longer than estimated walk times, parks were omitted with only entrances covered and an additional category was added to the advert types due to the presence of vinyl banners.

#### Data collection

Researchers were provided with a paper map of LSOA walking routes. Geo-located photographs of all eligible adverts within the LSOA boundary were taken via the researchers’ own smartphone cameras (smartphone models varied between researchers), with geolocation data collection enabled. Data collection was not carried out in public sites such as indoor shopping centres and indoor bus and train stations, as additional permissions are required to photograph in these locations. All adverts were captured (not just food and beverage adverts), providing an overall denominator for advertising exposure. Where the researcher encountered digital/mechanical advertisements that cycle through different advertising content, they ensured the whole cycle was captured.

#### Advertising assets in scope

Outdoor advertising assets may be owned by the local authority or privately owned – information which is not in the public domain. While HFAP are likely to extend only to council-owned advertising spaces, all outdoor advertisements meeting eligibility criteria were in scope for inclusion. Eligible outdoor advertising assets are defined as all intentionally placed permanent/semi-permanent advertisements that are visible from the street. Eligible advertising assets were informed by the Healthier Food Advertising Policy Toolkit^(22)^ with an additional ‘other’ category, in consultation with the project team, key stakeholders and other academics working in this field. Our list of fourteen eligible advertising asset types is illustrated in Table 1. Ineligible adverts were not photographed and are defined as unintentional, non-permanent or outside of the description of outdoor advertising. Examples include car parking or bus tickets, adverts on the insides or outsides of vehicles (e.g. buses, trains, taxis), litter, t-shirts, carrier bags, local authority websites and shop window signs.

**Table 1.** Eligible advertising assets for data collection

Type of eligible advertising	Image/description with examples
1. Bus shelters	Including digital bus shelter adverts
2. Other transport hubs	For example, tram and metro stations/stops
3. Large hoarding site	Usually found around construction work
4. Large digital sites	Digital billboards
5. Electronic free-standing displays	Known as 6 sheet panels
6. Non-electronic free-standing displays	Known as 4 sheet advertising. Often found in transport hubs
7. Smart benches	
8. Billboards	
9. Lamppost advertising ribbons/signs	
10. Totems	Free-standing columns covered with posters (no image available)
11. Litter and recycling bins	Public bins (no image available)
12. Signs	For example, roundabout signs and Welcome/boundary signs (no image available)
13. Telephone boxes	On the outside of telephone boxes, not including adverts on the inside (no image available)
14. Other	Other forms of outdoor advertising assets not captured. Examples include vinyl banners and posters

Images taken from Sustain and reproduced with permission^(22)^.

### Data processing

#### Attribute assignment

Advert images were coded against a range of attributes capturing location, advertising asset type and advert type. The full list of advert attributes, as well as metadata relating to these, is included in online supplementary material, Supplemental Table 4. Attribute coding was undertaken at four levels: image level (data collection date, LSOA identifier, longitude and latitude); asset level (asset ID, asset type, size (small, medium or large) and asset management company name); advert level (brand, product name, compliance); and product level (product type, price, weight, nutritional information, NPM score)^(30)^. Products were categorised into five groups: (1) food, (2) non-alcoholic beverage, (3) alcoholic beverage, (4) gambling or (5) other. The first four groups represent harmful forms of advertising that local authorities may wish to restrict, with the first two in the scope of the existing HFAP.

#### GPS location

GPS data were extracted from images using the Exif Tool Python package^(36)^, providing latitude and longitude coordinates for locations to be visualised on an interactive map^(37)^ (data dashboard). Forty-one images did not have embedded GPS coordinates, due to certain phone models storing image location at street address rather than the coordinate level (twenty-nine image ID) and unknown reasons (twelve image ID). To resolve this, coordinates from other images of the same asset were used (where available) or gathered from Google Street Maps.

#### Geographic exclusions

Advertising asset locations were mapped in QGIS prior to analysis to exclude adverts falling outside LSOA boundaries. These may have been captured by researchers due to human error, for example, misinterpretation of the map; equipment error, that is, inaccurate smartphone GPS location coding; and safety concerns, for example, taking photographs from afar where it was not possible to safely cross a busy road (e.g. advertisements on roundabouts).

#### Nutritional information

Product nutrition information was obtained per 100 g of product (enabling assignment of UK NPM scores) from manufacturer, restaurant or retailer websites. Where no nutrition information could be obtained, the closest match in the UK’s Food Tables CoFID^(38)^ was used or left blank where no close match could be identified. Where nutrition information was only available per portion of product (typical for out-of-home outlets), the product weight was sourced, enabling conversion to per 100 g. Data kindly shared by researchers at the University of Newcastle, who (between June 2022 and March 2023) manually weighed a sample of common items from large brands (e.g. McDonald’s and KFC)^(39)^, were first consulted for product weights. Where weights could not be found, values from the Food Standards Agency’s Food Portion Sizes handbook were used^(40)^ or left blank where there was no reasonable match. Nutritional coding was completed by the same person, so there was no need for inter-rater reliability tests. Where queries arose over the nutritional content of products, these were resolved in discussion with two or more members of the team.

#### Nutrient Profiling Model score estimation

The UK NPM score^(10)^ was calculated at the product level using the Consumer Data Research Centre’s NPM online calculator^(41)^ to assess its HFSS status (products failing the NPM = HFSS). Fruit, vegetable and nut (FVN) content is required as a percentage of total product weight for calculation of the NPM score. The FVN% was obtained from the ingredients list (where available) by summing the percentages of all fruits, vegetables and nuts listed in the ingredients. Where the ingredients list was unavailable or did not express the percentages of fruit, vegetable and nuts, CoFID data^(38)^ were searched for the closest matching product. Where no close match was found, the product was assumed to contain no fruit, vegetables or nuts, and a zero score was given for this domain. For adverts displaying several food items (e.g. a burger, fries and drink meal), each item was given an individual NPM score. If any product in an advert was considered HFSS, the advert was classified as ‘would be non-compliant’, that is, eligible for restriction under TfL’s HFAP rules. Food and drink brand advertisements (i.e. food brand logos/imagery where no food items are featured on the advert) were also classified as ‘would be non-compliant’.

### Analysis

#### Inter-rater reliability

Inter-rater reliability was calculated using Cohen’s kappa statistic to test for agreement in asset identification (two LSOA) and advert type coding (10 % of all images) by researcher 1 and researcher 2. The Cohen’s kappa statistic is used to calculate the probability of agreement being due to chance, as a measure of how reliably the data represent advertising exposure.

#### Advertising exposure

Absolute exposure to total advertising, food and non-alcoholic beverage advertising and would be non-compliant (HFSS and brand advertising combined) food and non-alcoholic beverage advertising was calculated at the LSOA level as a count and a proportion of all adverts observed. Absolute numbers and percentages across all LSOA are expressed at the IMD quintile level. *χ* ^2^ tests (at the 95 % confidence level) were used to assess the relationship between area deprivation and (1) total advertising exposure, (2) food and non-alcoholic beverage advertising exposure and (3) exposure to would be non-compliant food and non-alcoholic beverage adverts.

## Results

It was necessary to amend the planned visit order for the thirty LSOA (see online supplementary material, Supplemental Table 5), maintaining that LSOA from different quintiles were visited in each weekly block.

### Inter-rater reliability

In the two LSOA visited by two researchers (see online supplementary material, Supplemental Table 6), moderate agreement was observed for advertisement identification (Cohen’s kappa statistic = 0·65 estimated agreement = 0·71, *P* = 0·1557). Researchers were relatively likely to identify a different number of adverts as being ‘in scope’ with asset types: two (‘Litter and recycling bins’); eleven (‘Other transport hub’); and fourteen (‘Other’) contributing to disagreement. Where the asset type was included that was out of scope, this was resolved by a third researcher. For the 10 % sample of advert images which were dual coded, a 100 % agreement was found (see online supplementary material, Supplemental Table 7) (Cohen’s kappa = 1·00, estimated agreement = 1·00, *P* = 0·2234), showing that researchers were highly likely to agree on the asset type an advert belongs to.

### Descriptive statistics

Summary statistics for the thirty LSOA are presented by IMD quintile in Table 2. Overall, LSOA in IMD Q5 (least deprived) had the largest area (mean = 4·7 km^2^) and total network distance (mean = 44·74 km), reflecting lower population density. The central lines of major roads commonly form LSOA boundary lines. For completeness, advertising assessments on either side of the road were photographed and later excluded if they fell outside the LSOA boundary. This resulted in a total of 538 assets being photographed, with 243 assets excluded for being outside LSOA boundaries, leaving 295 outdoor advertising assets for analysis. Sensitivity analyses were performed for assets falling within 5, 10 and 20 metre buffers around LSOA boundary edges.

**Table 2.** Sample descriptive statistics by Index of Multiple Deprivation quintile of deprivation

	Total (*n* 30)			Q1 (*n* 6)			Q2 (*n* 6)			Q3 (*n* 6)			Q4 (*n* 6)			Q5 (*n* 6)		
	Total	Mean	sd	Total	Mean	sd	Total	Mean	sd	Total	Mean	sd	Total	Mean	sd	Total	Mean	sd
Sample area (km^2^) total, (mean, sd)	15·61	0·52	0·44	2·55	0·42	0·36	3·06	0·51	0·32	2·69	0·45	0·09	2·61	0·43	0·24	4·70	0·78	0·79
Network distance (km) total, (mean, sd)	193·31	6·44	2·81	38·82	6·47	3·39	37·01	6·17	1·68	36·91	6·15	0·73	35·83	5·97	2·92	44·74	7·46	3·84
	Count	Median	IQR	Count	Median	IQR	Count	Median	IQR	Count	Median	IQR	Count	Median	IQR	Count	Median	IQR
Total advertising assets – Count (median, IQR)	295	6·5	3·0–12·5	74	5·0	1·5–7·8	73	9·0	4·5–15·8	50	4·5	1·8–7·3	72	11·0,	5·3–15·3	26	5·0	1·8–9·8
	*n*	%		*n*	%		*n*	%		*n*	%		*n*	%		*n*	%	
Assets by type – Count (%)																		
Bus shelter	68	23·1		15	20·3		17	23·3		21	42·0		13	21·3		2	5·4	
Other transport hub	13	4·4		1	1·4		1	5·9		1	2·0		5	8·2		5	13·5	
Large hoarding site	24	8·1		16	21·6		2	2·7		2	4·0		4	6·6		0	0·0	
Large digital sites	5	1·7		1	1·4		2	2·7		1	2·0		1	1·6		0	0·0	
Electronic free-standing displays	3	1·0		1	1·4		1	5·9		0	0·0		1	1·6		0	0·0	
Non-electronic free-standing displays	39	13·2		5	6·8		6	8·2		6	12·0		11	18·0		11	29·7	
Smart benches	0	0·0		0	0·0		0	0·0		0	0·0		0	0·0		0	0·0	
Billboards	30	10·2		10	13·5		12	16·4		5	10·0		3	4·9		0	0·0	
Lamppost advertising ribbons	10	3·4		3	4·1		4	5·5		2	4·0		0	0·0		1	2·7	
Totems	9	3·1		2	2·7		2	2·7		0	0·0		4	6·6		1	2·7	
Litter and recycling bins	21	7·1		1	1·4		5	6·9		4	8·0		9	14·8		2	5·4	
Signs	24	8·1		13	17·6		5	6·9		4	8·0		2	3·3		0	0·0	
Telephone boxes	11	3·7		1	1·4		2	2·7		3	6·0		4	6·6		1	2·7	
Other	38	12·9		5	6·8		14	19·2		1	2·0		15	24·6		3	8·1	
Assets by size – Count (%)																		
Small	80	27·1		16	21·6		20	27·4		11	22·0		23	31·9		10	38·5	
Medium	156	52·9		31	41·9		36	49·3		31	62·0		42	58·3		16	61·5	
Large	59	20·0		27	36·5		17	23·3		8	16·0		7	9·7		0	0·0	
	Count	Median	IQR	Count	Median	IQR	Count	Median	IQR	Count	Median	IQR	Count	Median	IQR	Count	Median	IQR
Total adverts – Count (median, IQR)	437	8·0	3·0–15·3	99	5·5	1·5–8·0	90	9·5	6·8–19·8	53	4·5	1·8–7·3	129	11·0	5·3–39·3	66	10·0	3·0–14·8
	*n*	%		*n*	%		*n*	%		*n*	%		*n*	%		*n*	%	
Adverts by product type^*^ Count (%)																		
Food	67	15·1		17	17·0		23	24·5		10	18·5		9	7·0		8	12·1	
Non-alcoholic beverage	11	2·5		1	1·0		6	6·4		1	1·9		3	2·3		0	0·0	
Alcoholic beverage	23	5·2		6	6·0		3	3·2		7	13·0		5	3·9		2	3·0	
Gambling	0	0·0		0	0·0		0	0·0		0	0·0		0	0·0		0	0·0	
Other	342	77·2		76	76·0		62	66·0		36	66·7		112	86·8		56	84·9	
Food and drink adverts – Count^**^ (% of total adverts)	72	16·5		17	17·2		25	27·8		10	18·9		12	9·3		8	12·1	
HFSS status of food and non-alcoholic beverage adverts – Count (% of food and beverage adverts count)																		
HFSS	37	51·4		9	52·9		12	48·0		5	50·0		5	41·7		6	75·0	
Non-HFSS	24	33·3		5	29·4		9	36·0		5	50·0		4	33·3		1	12·5	
N/A^***^	11	15·9		3	17·7		4	16·0		0	0·0		3	25·0		1	12·5	
Would be non-compliant food and non-alcoholic beverage adverts – Count (% of total food and non-alcoholic beverage adverts)	48	66·7		12	70·6		16	64·0		5	50·0		8	66·7		7	87·5	

HFSS, high in fats, salt and sugars.

^*^Where a single advert features more than one product type, each product type is counted. Where an advert displays more than one product of the same type, the product type is counted once. ^**^The number of food and drink adverts refers to the total number of adverts featuring at least one food and/or non-alcoholic drink. The count may be less than the sum of the food and non-alcoholic beverages count by product type. ^***^N/A represents brand adverts or adverts which could not be assessed for Nutrient Profile Model status due to missing data.

#### Relationship between total advertising exposure and area deprivation

**Fig. 1:**
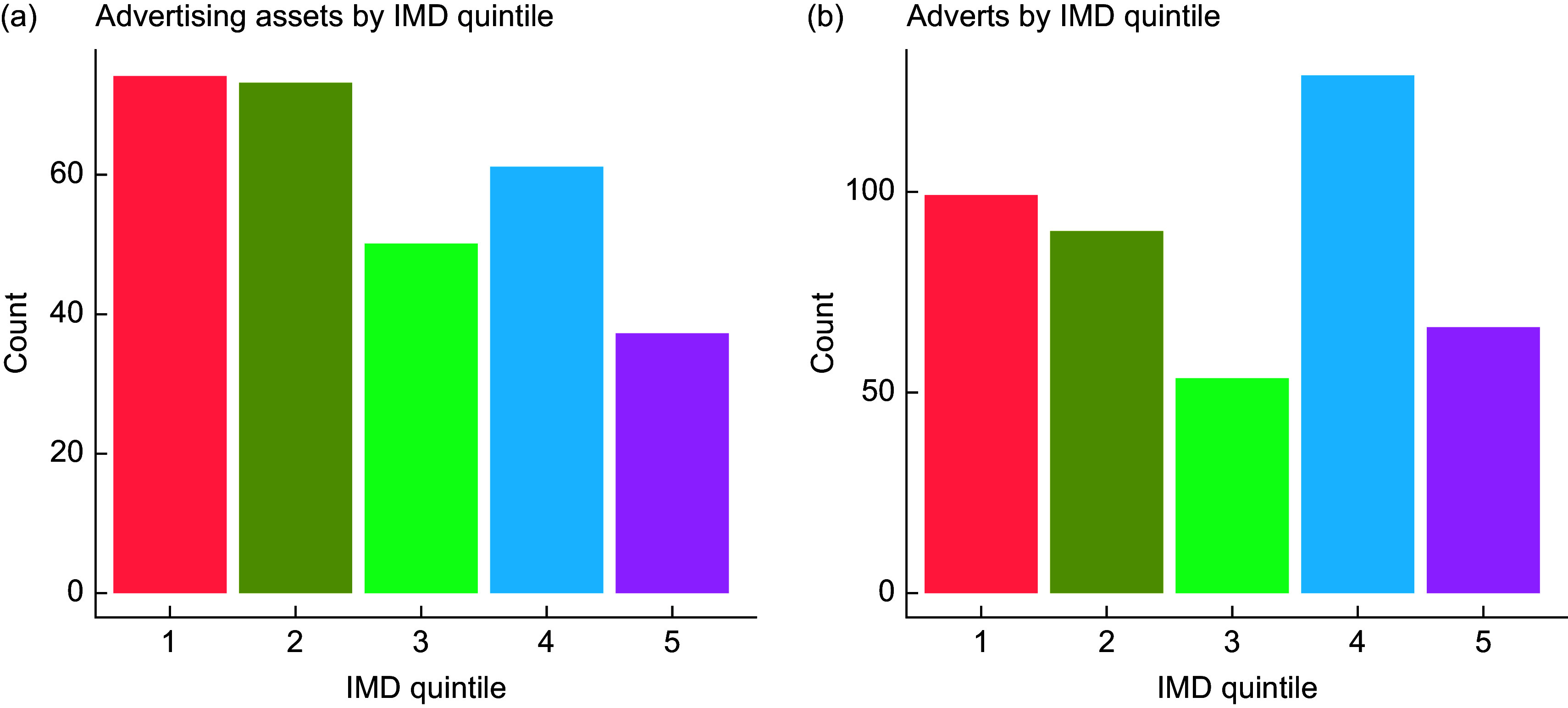
Bar chart showing number of advertising assets (a) and adverts (b) by Index of Multiple Deprivation (IMD) quintile.

**Fig. 2:**
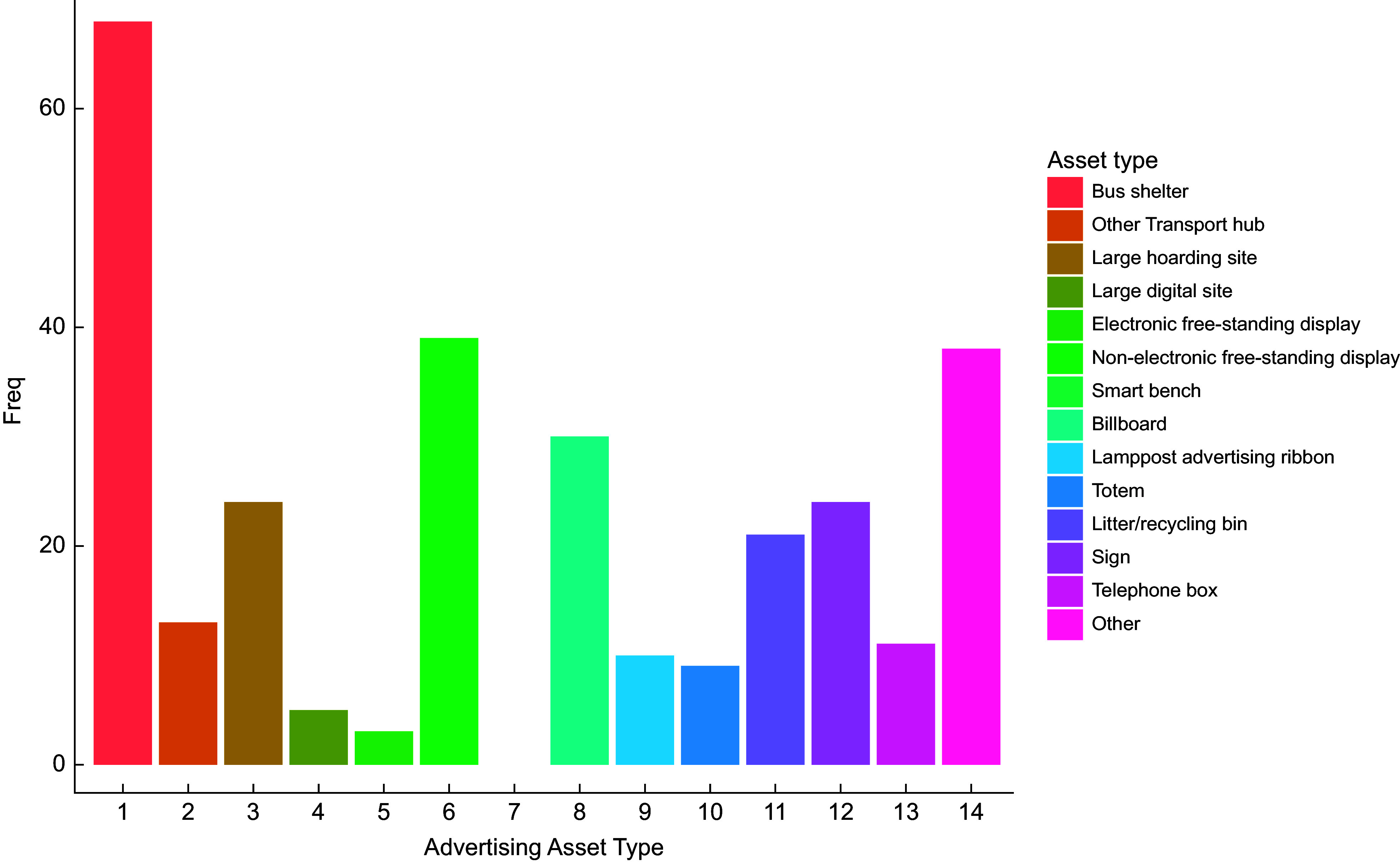
Bar chart showing all advertising assets by type (*n* 295).

The greatest number of advertising assets were found in Q1 – most deprived (*n* 74), Q2 (*n* 73) and Q4 (*n* 72) (Fig. 1). Q5 – least deprived, had the fewest assets (*n* 26), representing 9 % of all assets recorded. A breakdown of advertising assets by type can be observed in Fig. 2. Bus shelters were the most prevalent (*n* 68), representing 23 % of total assets. This was followed by non-electronic free-standing displays (*n* 39, 13 %). No smart benches were observed in the LSOA visited, and the number of electronic free-standing displays was small (*n* 3, 1 % of total assets). The highest prevalence of bus stop advertising assets was found in Q3 (*n* 21, 42 % of all assets in Q3), while Q5 recorded the lowest prevalence (*n* 2, 5 % of all Q5 advertising assets). Across all deprivation quintiles, outdoor advertising assets were most commonly of a medium size (*n* 156, 53 %). Q1 recorded the highest number of large advertising assets (billboards and large hoarding sites) *n* 27 (36 % of Q1 assets), while no large assets were found in Q5.

The 295 included advertising assets displayed 437 adverts (digital advertising assets and totems display more than one advert per asset). The highest number of adverts was found in Q4 (Fig. 1), which is likely to reflect the high prevalence of totems. The total amount of advertising does not appear to be evenly distributed by IMD quintile. In total, the 437 adverts displayed 684 products (a single advert may show more than one product, e.g. a pizza and a drink). ‘Other’ was the most common product type recorded (*n* 342, 77 % of all products). Sixty-seven food products (15 %), eleven non-alcoholic beverages (2 %) and twenty-three alcoholic beverages (5 %) were recorded. No gambling adverts were observed. Q2 had the highest number of food adverts (*n* 23, 24 % of all Q2 adverts), followed by Q1 (*n* 17, 17 % of Q1 adverts). Q4 had the lowest proportion of adverts displaying foods (7 %). Q3 had the highest number of alcoholic beverage adverts (*n* 7, 13 %).

#### Relationship between food and non-alcoholic beverage advertising exposure and area deprivation

**Fig. 3:**
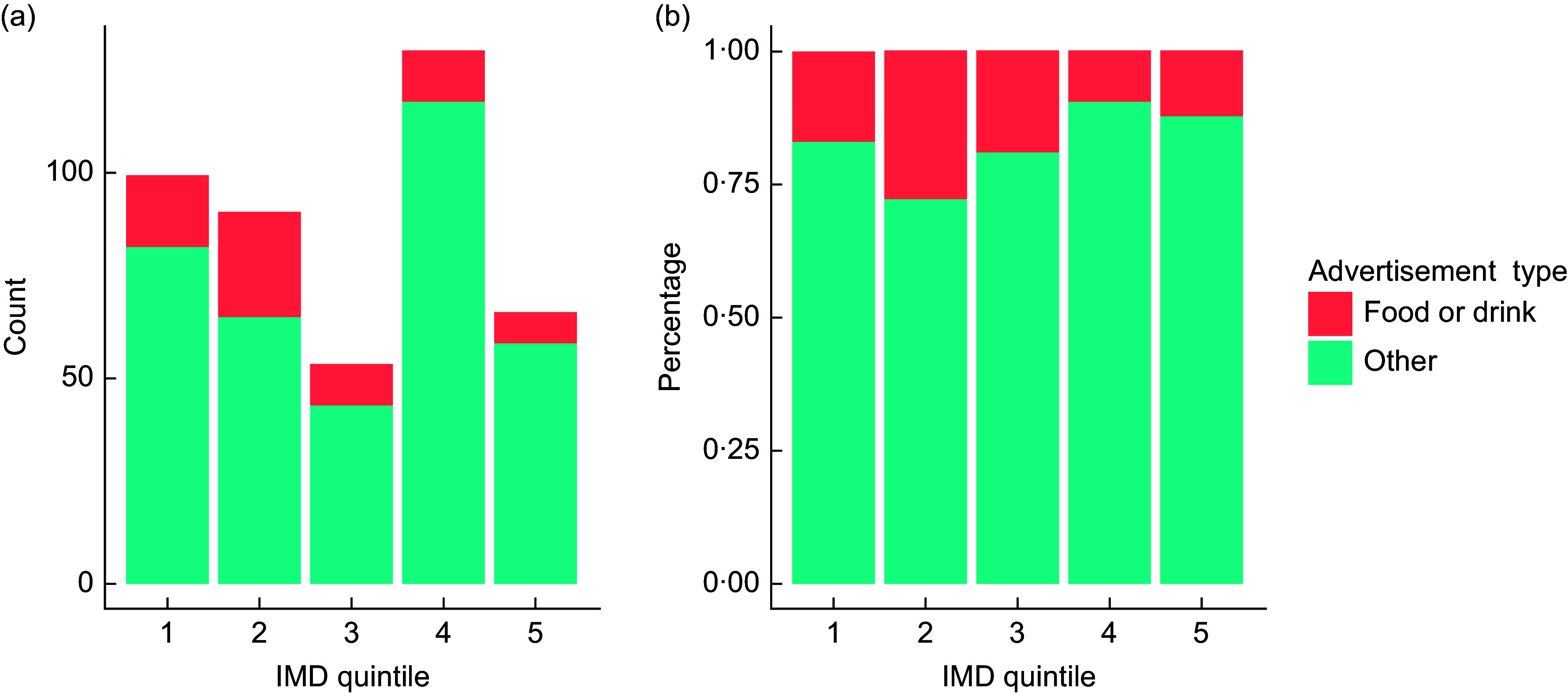
Stacked bar charts showing the number of food and drink adverts *v.* ‘other’ adverts by Index of Multiple Deprivation (IMD) quintile, expressed as counts (a) and percentage (b).

Food and drink adverts are counted as any advert displaying at least one food and/or non-alcoholic beverage product. In total, 72 food and drink adverts were recorded across the 30 Leeds LSOA, out of a total of 437 adverts. The remaining 365 adverts (84 %) display ‘other’ products that are not foods and/or non-alcoholic drinks. *χ* ^2^ analysis was used to explore the association between food and drink advertising exposure and area deprivation by testing the statistical significance of the difference in distribution (‘food and drink’ *v* ‘other’) across IMD quintiles. A statistically significant difference in the distribution of food and drink to other adverts by IMD quintile was found (*χ* ^2^ *P* = 0·006). Food and drink adverts represented a higher proportion of adverts in Q2 and a lower proportion in Qs 4 and 5 (least deprived) compared with what would be expected if there was no relationship (Fig. 3).

#### Relationship between exposure to would be non-compliant food and non-alcoholic beverage adverts and area deprivation

**Fig. 4:**
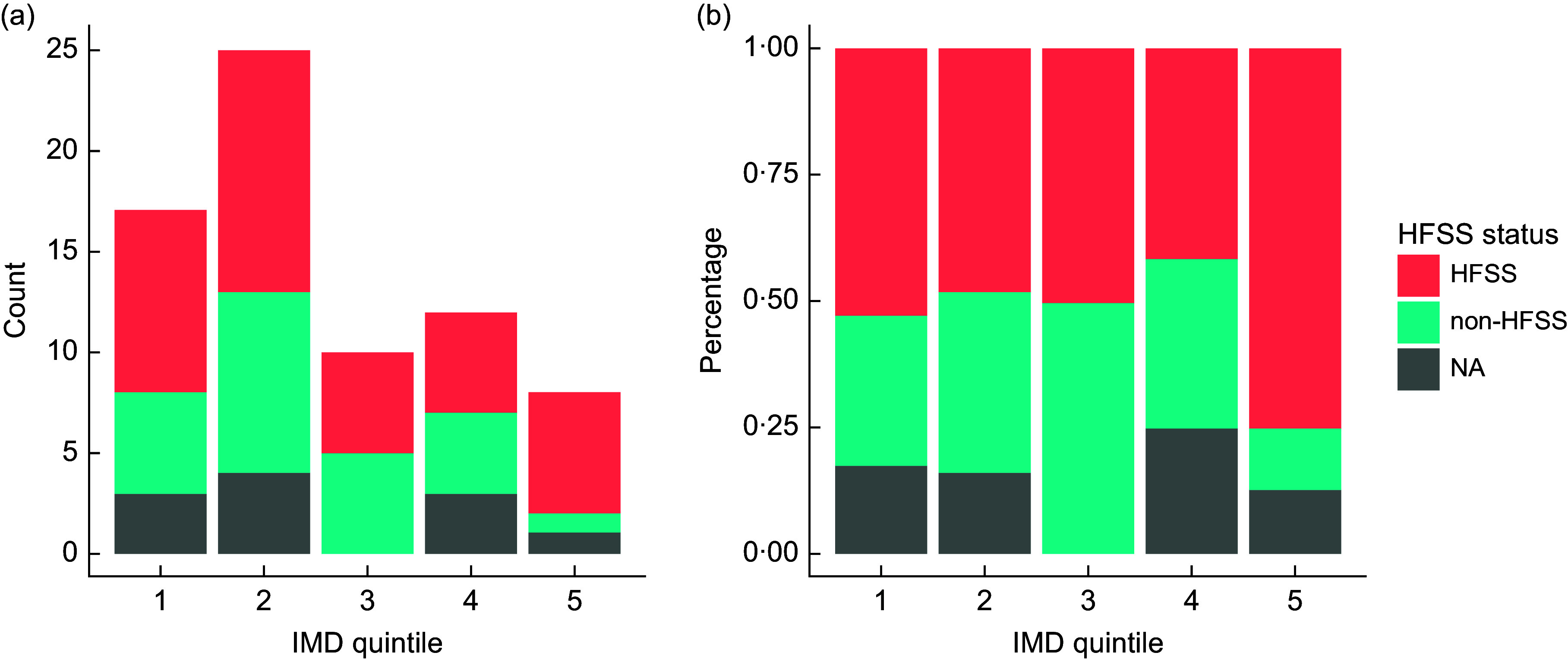
Stacked bar charts showing the number of food and drink adverts classed as high in fats, salt and sugars (HFSS), non-HFSS and ‘NA’, by Index of Multiple Deprivation (IMD) quintile, expressed as counts (a) and percentage (b).

**Fig. 5:**
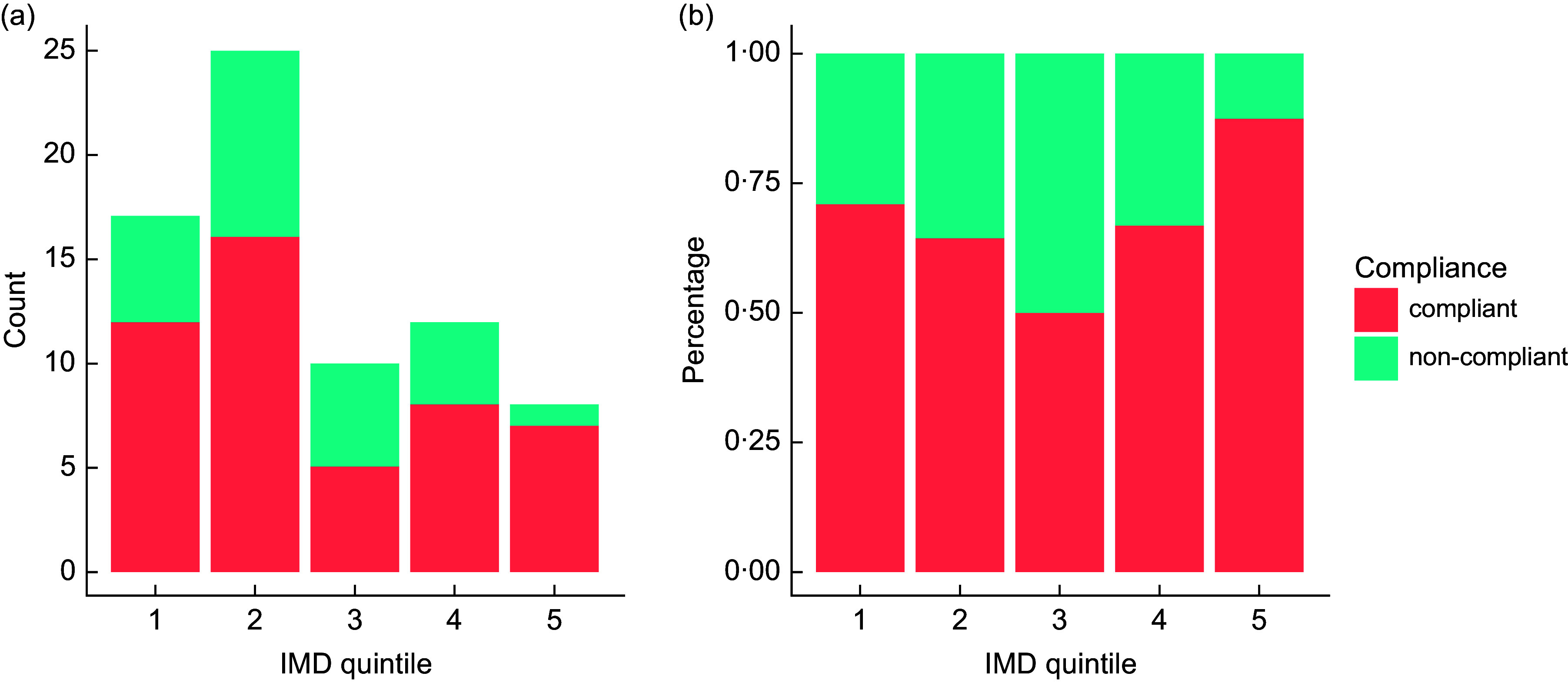
Stacked bar charts showing the number of food and drink adverts classed as compliant and non-compliant, by Index of Multiple Deprivation (IMD) quintile, expressed as counts (a) and percentage (b).

Due to low numbers of observations in each group, it was not possible to run a valid *χ* ^2^ analysis to test for statistical significance in the distribution of HFSS and non-HFSS food adverts by IMD quintile. N/A in Fig. 4 refers to food and non-alcoholic drink adverts that display brand advertising, or where insufficient data were available to assess a product’s HFSS status. While no formal statistical conclusion can be drawn, visual inspection of the data (Fig. 4) would suggest that there is a higher proportion of HFSS food and drink adverts in Q5, but counts are low, with only eight food and drink adverts found in Q5 in total. More extensive data collection is required to produce reliable results on the association between HFSS advert status and IMD quintile. Likewise, for the advert compliance analysis (Fig. 5), numbers of observations were too low in some areas to form meaningful conclusions about the relationship between advert compliance and IMD quintile.

Sensitivity analysis for LSOA exclusion buffers revealed no differences in observed patterns, and sample sizes remained too small for results to be meaningful for tests for variation in HFSS status and advert compliance by IMD quintile.

**Fig. 6:**
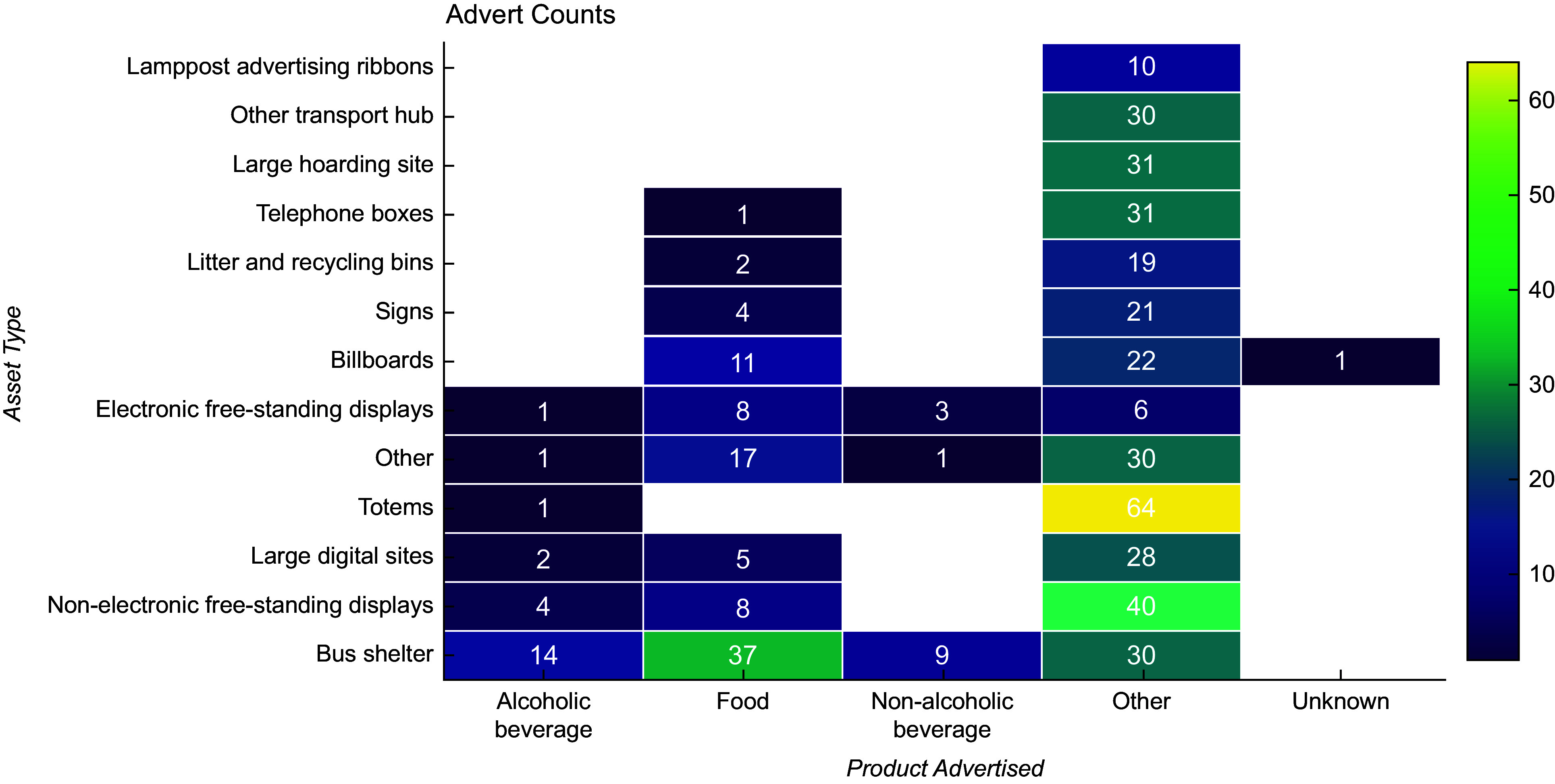
Heat map of advert counts by advert product type and advertising asset type.

While the greatest number of adverts were found on totems (*n* 65), they are dominated by ‘other’ products (*n* 64) and did not display any food or non-alcoholic beverage adverts (Fig. 6). Bus shelters display the greatest number of food (*n* 37), non-alcoholic beverage (*n* 9) and alcoholic beverage adverts (*n* 14). Other assets (*n* 17), billboards (*n* 11), electronic free-standing displays (*n* 8) and non-electronic free-standing displays (*n* 8) are also common sources of food advertising.

## Discussion

This study identified 295 outdoor advertising assets across thirty LSOA in Leeds, displaying 437 adverts. A quarter of advertising assets recorded were in the highest quintile of deprivation and just 9 % in the least deprived quintile. Such a relationship between outdoor advertising prevalence and deprivation has been reported elsewhere^(42,43)^. Our results also suggest that the relationship between advertising prevalence and area deprivation is non-linear. We found a similar number of adverts in IMD quintiles 1 (most deprived), 2 and 4, though this is driven by asset type with totems displaying ‘other’ products, such as music and event adverts accounting for a large number of quintile four adverts.

Our study found 15 % of all advertisements in sampled areas of Leeds to be for food and drink products, with just over half of these classified as HFSS (‘unhealthy’). A further 17 % of food and drink adverts displayed brand-only advertising, meaning 67 % of the food and non-alcoholic beverage adverts observed in Leeds would be considered non-compliant under TfL-based HFAP guidelines. In Newcastle, Adams *et al*.^(43)^ observed a similar percentage of overall advertising space dedicated to food and drink, but a smaller proportion (one-third) was classified as HFSS compared with the Leeds sample, with no brand-only food and drink adverts observed^(43)^. These findings may reflect spatial differences in food and drink advertising exposures between UK cities or emerging temporal trends in HFSS marketing over the intervening 12 years (2011–2023). A possible unintended consequence of television, digital media and in-store HFSS marketing restrictions may have been to encourage brands to displace HFSS advertisements to outdoor advertising spaces. More regular data collection across the UK would be required to uncover emergent trends in outdoor advertising.

The relationship between area socio-economic status and area demographic characteristics and exposure to food and drink advertising is inconsistent. For example, a study in Liverpool observed 58 % of food and drink advertising was in the two most deprived deciles^(35)^, yet in the North-East and Scotland, there was no social patterning of unhealthy advertising on bus shelters^(24,39)^.

In our study, we observed a non-linear relationship between area demographic patterning of food and drink advert distributions which suggest that built environment factors are also important determinants of advertising prevalence. For example, the Q2 areas (where food and drink advertising prevalence were highest) sampled in our study were typically larger in size and characterised by intersections with main roads and high streets, while Q1 areas were largely residential. Self-reported data across the UK have shown that more vulnerable groups, including lower socio-economic groups and younger individuals, reported seeing a greater level of unhealthy product advertising in their local neighbourhood, predominantly for fast food products^(44,45)^. In line with our findings on advert locations, self-reported exposure in Bristol and South Gloucestershire was highest in deciles 3–4 (equivalent to quintile 2), with 67 % of individuals recalling HFSS exposure within the previous week^(45)^, followed by deciles 1–2 (equivalent to quintile 1). Interestingly, Q2 (equivalent to decile 3–4) also contained the greatest number of unhealthy adverts in our study. Mobility and built environment data are likely key to better understanding exposures to outdoor advertising^(22)^.

Bus shelters are the most prevalent form of outdoor advertising asset in Leeds, displaying 23 % of all adverts and 40 % of food adverts, of which 26 % were HFSS foods. These findings are similar to those from other northern UK cities, including in Middlesbrough, Redcar and Cleveland (in the North East of England) where half of all bus shelter advertisements were for food and drink products, 35 % of which were HFSS^(39)^, and in Scotland where a third of bus shelter adverts fell into unhealthy food categories (according to researcher-defined classification)^(25)^. However, evidence from Bristol and South Gloucestershire showed food/drink advertising to account for a much lower proportion of total bus shelter advertising (24 %), just 12 % of which were HFSS^(46)^, which may suggest a North-South divide with bus shelters contributing more to unhealthy food advertising exposures in the North.

With advertising exposure increasing the likelihood of consuming the advertised product (particularly for fast food, sugary drinks and sugary cereals)^(46)^, the impact of outdoor marketing on dietary behaviours could be substantial. Indeed, the TfL HFAP has demonstrated a significant decline in purchased calories from HFSS categories, but further evaluation of HFAP in other local councils would build a more robust evidence base.

Beyond food advertising, other research has found harmful commodities, such as alcohol and gambling, to be commonplace on outdoor advertising assets^(47)^. But this was not the case in our study. No gambling adverts were found within our visited areas of Leeds, and just twenty-three alcohol adverts were observed, too small a sample to assess the relationship with area deprivation.

To date more than 80 of the 317 local authorities across England have expressed an interest in adopting a HFAP^(48)^, with 12 local authorities having active policies in place at the time of writing^(49)^. Despite apparent will, HFAP development can be slowed by political complexities including industry lobbying and fears over advertising revenue losses for local authorities. Although evidence from London suggests concerns for advertising revenue may be unwarranted, with TfL experiencing a £2·3 million increase in advertising revenue within the first year of the HFAP^(50)^, budgetary arguments can be hard to overcome. Robust localised evidence on the current outdoor advertising environment and understanding of associations with health outcomes is therefore needed^(17)^.

### Strengths

Our study sampled an equal number of LSOA from each deprivation quintile, enabling robust comparisons between area deprivation and outdoor advertising exposure. By collecting data at the LSOA level, we generate findings which are meaningful from a policy perspective and enable area-level linkage with other population statistics. By gathering data on all outdoor advertising asset types, we could reveal the asset types most commonly displaying unhealthy food and drink products, highlighting priorities for the scope of HFAP. Classifying products by the NPM enabled comparison with current HFSS legislation. Furthermore, by capturing our data within a defined timeframe, we avoided repeated advert cycles and seasonal variations in advertising.

### Limitations

The choice of LSOA neighbourhoods as the geography for sampling advertising assets introduces some limitations. LSOA boundaries are atypical in shape, often running down the middle of roads. LSOA are administrative geographies and are therefore unlikely to represent how residents move around their neighbourhoods and do not offer a full view of outdoor advertising exposure. Furthermore, LSOA vary in size due to their population weighted nature, meaning it is not possible to easily compare the relative geographical density of adverts in each area (although it does enable population weighted density which is desirable).

Assessment of inter-rater reliability revealed ambiguity in interpretation of which adverts were in scope for the study during fieldwork. Some asset types (the ‘other’ category) were particularly ambiguous suggesting that even more detailed definition might be useful. Knowledge of which assets are owned by local authorities (*v* privately owned) would be beneficial for assessing compliance with and potential impacts of a HFAP.

In-person auditing of LSOA limited coverage of the city compared with other studies which utilised secondary data (e.g. Google Street View). Nonetheless, our study avoided limitations of Google Street View such as missing data, lack of temporal consistency and inability to control for advert cycles, as images may be captured over prolonged periods and on different dates.

### Future research and policy implications

Future work may consider standardising approaches to geographic data sampling for improved comparability and the incorporation of transport network and GPS traces from mobile phones or footfall sensor data to account for how people move around as a factor in their advertising exposures.

Sustainability of advertised products should also be investigated, given HFSS products tend to be less sustainable.

Open data on outdoor food advertising in the UK are lacking, limiting opportunities to understand the harms that outdoor advertising may be doing in local communities and the potential for HFAP as a protective public health measure. Scalable data collection methods enabling ongoing longitudinal monitoring and research into outdoor advertising should be considered. Approaches may include secondary data sources (such as those held by local authorities) or citizen science.

### Conclusions

In conclusion, findings demonstrate non-linear inequalities in the advertising of food and drink adverts across levels of deprivation in Leeds, with an indication towards more HFSS adverts in more deprived areas. Bus shelters were the most likely advertising asset to display unhealthy food and drink adverts, indicating an important target for HFAP. We advocate for longitudinal data collection to reveal trends in advertising exposures and assessment of success and compliance with advertising policies.

## Supporting information

Jenneson et al. supplementary material Jenneson et al. supplementary material

## Supplementary material

For supplementary material accompanying this paper, visit https://doi.org/10.1017/S1368980025100670.
