# Supplementary material for: Mapping Advertising Assets Project: a cross-sectional analysis of food-related outdoor advertising and the relationship with deprivation in Leeds, UK
Source: Public Health Nutr. 2025 Sep 3;28(1):e161. doi: 10.1017/S1368980025100670 (PMC12516639; doi:10.1017/S1368980025100670)
Supplement: Jenneson et al. supplementary material [file S1368980025100670sup001.docx]

Supplementary Table 1. Final list of 30 LSOAs included in the data collection, with an equal distribution of 6 from each quintile

| **LSOA Identifier** | **LSOA Name** | **Ward** | **Leeds IMD rank** | **IMD Quintile** |
| --- | --- | --- | --- | --- |
| E01011372 | Stratford Street, Beverleys | Hunslet & Riverside (HR) | 1 | 1 |
| E01011662 | Foundry Mill Terr, Brooklands | Killingbeck & Seacroft (KS) | 2 | 1 |
| E01011368 | Crosby St, Recreations, Bartons | Beeston & Holbeck (BH) | 3 | 1 |
| E01011363 | Holdforths, Clyde Approach, | Armley (AY) | 4 | 1 |
| E01011625 | St Hildas, Copperfields, Gartons | Burmantofts & Richmond Hill (BR) | 6 | 1 |
| E01011427 | Easterly Grove, St Wilfrids | Gipton & Harehills (GH) | 12 | 1 |
| E01033021 | Upper Accommodation Rd… | Burmantofts & Richmond Hill (BR) | 103 | 2 |
| E01011726 | Gamble Lane, Tong Drive, Stonecliffes, Hall Lane | Farnley & Wortley (FW) | 118 | 2 |
| E01011448 | Hyde Park Road, Royal Park Road, Brudenell St | Headingley & Hyde Park (HH) | 148 | 2 |
| E01011341 | Veritys, Dunhills, | Temple Newsam (TN) | 149 | 2 |
| E01011319 | Barkly Road, Allenbys, Athas | Beeston & Holbeck (BH) | 189 | 2 |
| E01011597 | Galloway Lane, Moorland Rd, Ederoyd Cres | Calverley & Farsley (CF) | 191 | 2 |
| E01011267 | West Queensway, Greenbottom | Guiseley & Rawdon (GR) | 195 | 3 |
| E01011477 | Eden Cres, Beecroft St, Sandford Rd | Kirkstall (KL) | 205 | 3 |
| E01032489 | Albert Rd, Denshaw Dr, Troy Rd | Morley South (MS) | 210 | 3 |
| E01011287 | Spring Valleys | Armley (AY) | 214 | 3 |
| E01011388 | Cookridge Hospital, Haven Chase, Eaton Hill | Weetwood (WW) | 264 | 3 |
| E01011651 | Birchwood Hill, West Park Dr, Roman Ave | Alwoodley (AL) | 283 | 3 |
| E01011445 | Pearson Grove, Chestnut Road, Welton Mount | Headingley & Hyde Park (HH) | 318 | 4 |
| E01011383 | Raynels, The Drive | Adel & Wharfedale (AW) | 329 | 4 |
| E01011329 | Calverleys, Cambridge Gardens, Whitecote Rise | Bramley & Stanningley (BS) | 337 | 4 |
| E01011642 | Glehow Lane, Gledhow, Wood Rd, Well House Rd | Roundhay (RH) | 345 | 4 |
| E01032496 | Robin Hood | Ardsley & Robin Hood (AR) | 382 | 4 |
| E01033015 | Granary Wrf, Whitehall Waterfront… | Hunslet & Riverside (HR) | 385 | 4 |
| E01011605 | Windmill Hill | Pudsey (PY) | 393 | 5 |
| E01011694 | Becketts Parks, St Chads | Weetwood (WW) | 398 | 5 |
| E01011522 | Gildersome Town St, Highfield Close | Mrley North (MN) | 408 | 5 |
| E01011397 | Meadow Road / Firthfields (East Garforth) | Garforth & Swillington (GS) | 422 | 5 |
| E01011353 | Montreal Ave, Gledhow Parks, Roxholmes | Chapel Allerton (CA) | 435 | 5 |
| E01011271 | Fieldhead, Hawkstones | Guiseley & Rawdon (GR) | 444 | 5 |

Supplementary Table 2. Leeds LSOAs within most deprived 1% nationally, according to IMD 2019 rank

| **LSOA Identifier** | **LSOA name** | **Priority ward** |
| --- | --- | --- |
| E01011372 | Stratford Street, Beverleys | Hunslet & Riverside |
| E01011662 | Foundry Mill Terr, Brooklands | Killingbeck & Seacroft |
| E01011368 | Crosby St, Recreations, Bartons | Beeston & Holbeck |
| E01011363 | Holdforths, Clyde Approach, | Armley |
| E01011375 | Wickham St. Seftons, Harlechs | Hunslet & Riverside |
| E01011625 | St Hildas, Copperfields, Gartons | Burtmantofts & Richmond Hill |
| E01011658 | Boggart Hill | Killingbeck & Seacroft |
| E01011667 | Foundry Mill Drive, Hawkshead Cres, Alston Lane | Killingbeck & Seacroft |
| E01011347 | Cliftons, Nowells | Burtmantofts & Richmond Hill |
| E01011294 | Armley Grove Place, Hall Lane, Abbot View | Armley |
| E01011623 | East Park Drive, Glensdales, Raincliffes | Burtmantofts & Richmond Hill |
| E01011427 | Easterly Grove, St Wilfrids | Gipton & Harehills |

Areas highlighted in yellow were selected for data collection due to their priority status (highest IMD ranking at the ward-level).

Supplementary Table 3. Allocated visit order for LSOAs, determined by block random sampling

| **LSOA** | **Quintile** | **Allocated visit order** |
| --- | --- | --- |
| E01011662 | 1 | 1 |
| E01011448 | 2 | 2 |
| E01011267 | 3 | 3 |
| E01032496 | 4 | 4 |
| E01011271 | 5 | 5 |
| E01011427 | 1 | 6 |
| E01011597 | 2 | 7 |
| E01032489 | 3 | 8 |
| E01011383 | 4 | 9 |
| E01011605 | 5 | 10 |
| E01011363 | 1 | 11 |
| E01033021 | 2 | 12 |
| E01011287 | 3 | 13 |
| E01011445 | 4 | 14 |
| E01011694 | 5 | 15 |
| E01011372 | 1 | 16 |
| E01011319 | 2 | 17 |
| E01011477 | 3 | 18 |
| E01011642 | 4 | 19 |
| E01011397 | 5 | 20 |
| E01011625 | 1 | 21 |
| E01011726 | 2 | 22 |
| E01011388 | 3 | 23 |
| E01033015 | 4 | 24 |
| E01011522 | 5 | 25 |
| E01011367 | 1 | 26 |
| E01011341 | 2 | 27 |
| E01011651 | 3 | 28 |
| E01011329 | 4 | 29 |
| E01011353 | 5 | 30 |

Supplementary Table 4. Advert attributes for image coding

| **Code** | **Attribute** | **Meta-data** |
| --- | --- | --- |
| Rinitials | Researcher Initials | Initials of the researcher who carried out the data collection |
| ASSETid | Asset identifier | Unique advertising asset id assigned by order of data capture. A single advertising asset may be associated with more than one advertisement, in the case of digital displays and totems, which contain more than one advertisement.  Nomenclature format = string comprising of 2 letter Ward identifier followed by 4-letter LSOA id (see appendix 1) and number in order of asset collection starting at 01  e.g. HR_SSBY_01 would be the first advertising asset collected for Hunslet & Riverside, Stratford Street, Beverleys |
| ADid | Advertisement identifier | A unique advert on an advertising asset. More than one advert may be associated with a single advertising asset e.g. in the case of digital displays and totems.  Similar nomenclature to ASSETid but with an additional two-number identifier ordered from 01  e.g. HR_SSBY_01_01 would be the first advert on the first advertising asset collected for Hunslet & Riverside, Stratford Street, Beverleys |
| IMGid | Image identifer | Unique image id assigned by phone/device  One image per advertisement is ideal, though in some cases more than one image may be needed to capture full advert detail. |
| IMGdate | Date | Date of image capture in format DD/MM/YYYY |
| LSOA | LSOA identifier | 11-digit LSOA identifier beginning E01 |
| Long | Longitude | X – coordinate, determined from GPS location from EXIF image data |
| Lat | Latitude | Y – coordinate, determined from GPS location from EXIF image data |
| ADtype | Advertising asset type | Coded numerically, 1 – 14  1 – Bus shelter  2 – Other transport hub  3 - Large hoarding site  4 - Large digital sites  5 - Electronic free-standing displays  6 - Non-electronic free-standing displays  7 - Smart benches  8 - Billboards  9 - Lamppost advertising ribbons  10 - Totems  11 - Litter and recycling bins  12 - Signs  13 - Telephone boxes  14 - Other |
| ADsize | Advert size | 1 = small (advertising types 9, 11, 12)  2 = medium (advertising types 1, 2, 5, 6, 7, 10, 13)  3 = large (advertising types 3, 4, 8) |
| ADprodtype | Advertised product type | 1 = food  2 = non-alcoholic beverage  3 = alcoholic beverage  4 = gambling  5 = other |
| ADbrand | Brand | Name of brand being advertised |
| ADprod | Product name | Name of product being advertised. In the case of brand advertising where no particular product is being advertised, leave as NA  Where more than one product included in an advert (e.g. adverts showing a whole meal), a separate row should be created for each product – product-level nutrition information can then be aggregated at the advert level |
| Brandad | Brand advertisement | 1 = brand advertising  0 = not brand advertising |
| ADmanagement | Asset management company name | Name of company that manages the advertising asset (e.g., clear channel) |
| Cinitials | Coder initials | Initials of researcher undertaking data coding |
| Codedate | Code date | Date of data coding |
| Price | Product price (£) | Price of product on the day of coding (according to internet searches), preferably from manufacturer’s website – where price cannot be obtained from manufacturer’s website, use price on Tesco website, UK’s largest grocery retailer. Where no price can be obtained, leave blank.  In the case of brand advertising where no particular product is being advertised, leave blank. |
| Pricesourcelink | Link to source of price information | Where appropriate (manufacturer, restaurant or retailer website) include the url from where priceinformation was obtained. The access date should be the same as the coding date. |
| Unit | Weight/volume unit | g = grams  ml = millilitres |
| Portion | Portion size | Portion weight (g) or volume (ml) as indicated by Unit column. This is the amount of product intended to be consumed in a single sitting by one person, according to the brand. |
| Totalwt | Total weight/volume | The total amount of food being shown in the advert e.g. a 2 litre bottle of soft drink would have a total weight of 2000ml but the portion amount would be smaller. |
| Wtsource | Source of weight information | Include url if from retailer/manufacturer website, reference to page in FSA book, or Scott image data |
| Nutritionsource | Source of nutritional information | 0 = no nutritional information available  1 = manufacturer website  2 = restaurant website  3 = retailer website  4 = CoFID tables |
| Nutsourcelink | Link to nutrition information source | Where appropriate (manufacturer, restaurant or retailer website) include the url from where nutrition information was obtained. The access date should be the same as the coding date. |
| Fatdensity | Total fat density | g/100g of product |
| Satfatdensity | Saturated fat | g/100g of product |
| Sugardensity | Total sugars | g/100g of product |
| Sodiumdensity | Total sodium | mg/100g of product |
| Ekcaldensity | Energy | Kcal/100g of product |
| Fibredensity | Fibre (as AOAC) | g/100g of product |
| Proteindensity | Protein | g/100g of product |
| FVN | Fruit, vegetable and nut % | Numeric value from 0 – 100, representing the % of product made up of fruit, vegetables and/or nuts |
| NPMscore | UK NPM score | Numeric value (can include negative values) |
| NPMstatus | UK NPM status | 0 = pass  1 = fail |
| ADcompliance | Advert compliance with proposed healthy advertising policy restrictions | 0 = compliant  1 = non-compliant  Where any food on the advert is classed as HFSS (I.e. fails the NPM) the advert will be deemed non-compliant.  Where the advert is for brand advertising only, it will be deemed non-compliant. |

Supplementary Table 5. Visit dates for LSOAs, comparing actual with allocated visit order.

| **Date** | **Allocated visit order** | **Actual visit order** | **LSOA** | **Quintile** |
| --- | --- | --- | --- | --- |
| 11^th^ May | 7 | 1 | E01011597 | 2 |
| 11^th^ May | 10 | 2 | E01011605 | 5 |
| 15^th^ May | 4 | 3 | E01032496 | 4 |
| 16^th^ May | 5 | 4 | E01011271 | 5 |
| 17^th^ May | 8 | 5 | E01032489 | 3 |
| 18^th^ May | 3 | 6 | E01011267 | 3 |
| 18^th^ May | 6 | 7 | E01011427 | 1 |
| 18^th^ May | 1 | 8 | E01011662 | 1 |
| 19^th^ May | 13 | 9 | E01011287 | 3 |
| 19^th^ May | 11 | 10 | E01011363 | 1 |
| 19^th^ May | 15 | 11 | E01011694 | 5 |
| 22^nd^ May | 14 | 12 | E01011445 | 4 |
| 22^nd^ May | 2 | 13 | E01011448 | 2 |
| 23^rd^ May | 9 | 14 | E01011383 | 4 |
| 23^rd^ May | 20 | 15 | E01011397 | 5 |
| 23^rd^ May | 12 | 16 | E01033021 | 2 |
| 24^th^ May | 18 | 17 | E01011477 | 3 |
| 24^th^ May |  |  | E01011694 (second researcher) | 5 |
| 25^th^ May | 16 | 18 | E01011372 | 1 |
| 25^th^ May | 21 | 19 | E01011625 | 1 |
| 25^th^ May | 24 | 20 | E01033015 | 4 |
| 1^st^ June | 23 | 21 | E01011388 | 3 |
| 1^st^ June | 19 | 22 | E01011642 | 4 |
| 5^th^ June | 25 | 23 | E01011522 | 4 |
| 6^th^ June | 17 | 24 | E01011319 | 2 |
| 6^th^ June | 26 | 25 | E01011367 | 1 |
| 7^th^ June | 29 | 26 | E01011329 | 4 |
| 7^th^ June | 22 | 27 | E01011726 | 2 |
| 9th June | 27 | 28 | E01011341 | 2 |
| 12^th^ June |  |  | E01011642 (second researcher) | 4 |
| 15^th^ June | 30 | 29 | E01011353 | 5 |
| 15^th^ June |  |  | E01011397 (second researcher) | 5 |
| 15^th^ June | 28 | 30 | E01011651 | 3 |

*Different shades of grey distinguish between weekly blocks.*

Supplementary Table 6. Inter-rater reliability for advert identification (n=3 LSOAs)

|  | **Rater 2** | **Asset type** | | | |  |  |  |  |  |  |  |  |  |  |
| --- | --- | --- | --- | --- | --- | --- | --- | --- | --- | --- | --- | --- | --- | --- | --- |
| **Rater 1** | **1** | **2** | **3** | **4** | **5** | **6** | **7** | **8** | **9** | **10** | **11** | **12** | **13** | **14** | **Total** |
| **Asset type** |  |  |  |  |  |  |  |  |  |  |  |  |  |  |  |
| **1** | 8 |  |  |  |  |  |  |  |  |  |  |  |  |  | 8 |
| **2** |  | **3** |  |  |  |  |  |  |  |  |  |  |  |  | 7 |
| **3** |  |  | 0 |  |  |  |  |  |  |  |  |  |  |  | 0 |
| **4** |  |  |  | 0 |  |  |  |  |  |  |  |  |  |  | 0 |
| **5** |  |  |  |  | 0 |  |  |  |  |  |  |  |  |  | 0 |
| **6** |  |  |  |  |  | 16 |  |  |  |  |  |  |  |  | 16 |
| **7** |  |  |  |  |  |  | 0 |  |  |  |  |  |  |  | 0 |
| **8** |  |  |  |  |  |  |  | 0 |  |  |  |  |  |  | 0 |
| **9** |  |  |  |  |  |  |  |  | 0 |  |  |  |  |  | 0 |
| **10** |  |  |  |  |  |  |  |  |  | 1 |  |  |  |  | 1 |
| **11** |  |  |  |  |  |  |  |  |  |  | **4** |  |  |  | 4 |
| **12** |  |  |  |  |  |  |  |  |  |  |  | 0 |  |  | 0 |
| **13** |  |  |  |  |  |  |  |  |  |  |  |  | 1 |  | 1 |
| **14** |  |  |  |  |  |  |  |  |  |  |  |  |  | **3** | 14 |
| **Total** | 8 | 3 | 0 | 0 | 0 | 16 | 0 | 0 | 0 | 1 | 5 | 0 | 1 | 3 | 51 |

*Numbers represent the total assets recorded by each researcher under each of the 14 asset types. Numbers highlighted in bold show instances where raters did not agree.*

Supplementary Table 7. Inter-rater reliability for asset type coding (10% total advert data)

|  | **Rater 2** | **Asset type** | | | |  |  |  |  |  |  |  |  |  |  |
| --- | --- | --- | --- | --- | --- | --- | --- | --- | --- | --- | --- | --- | --- | --- | --- |
| **Rater 1** | **1** | **2** | **3** | **4** | **5** | **6** | **7** | **8** | **9** | **10** | **11** | **12** | **13** | **14** | **Total** |
| **Asset type** |  |  |  |  |  |  |  |  |  |  |  |  |  |  |  |
| **1** | 10 |  |  |  |  |  |  |  |  |  |  |  |  |  | 10 |
| **2** |  | 0 |  |  |  |  |  |  |  |  |  |  |  |  | 0 |
| **3** |  |  | 0 |  |  |  |  |  |  |  |  |  |  |  | 0 |
| **4** |  |  |  | 15 |  |  |  |  |  |  |  |  |  |  | 15 |
| **5** |  |  |  |  | 3 |  |  |  |  |  |  |  |  |  | 3 |
| **6** |  |  |  |  |  | 2 |  |  |  |  |  |  |  |  | 2 |
| **7** |  |  |  |  |  |  | 0 |  |  |  |  |  |  |  | 0 |
| **8** |  |  |  |  |  |  |  | 5 |  |  |  |  |  |  | 5 |
| **9** |  |  |  |  |  |  |  |  | 0 |  |  |  |  |  | 0 |
| **10** |  |  |  |  |  |  |  |  |  | 22 |  |  |  |  | 22 |
| **11** |  |  |  |  |  |  |  |  |  |  | 0 |  |  |  | 0 |
| **12** |  |  |  |  |  |  |  |  |  |  |  | 27 |  |  | 27 |
| **13** |  |  |  |  |  |  |  |  |  |  |  |  | 0 |  | 0 |
| **14** |  |  |  |  |  |  |  |  |  |  |  |  |  | 0 | 0 |
| **Total** | 10 | 0 | 0 | 15 | 3 | 2 | 0 | 5 | 0 | 22 | 0 | 27 | 0 | 0 | 84 |

*Numbers represent the total assets recorded by each researcher under each of the 14 asset types. Numbers highlighted in bold show instances where raters did not agree.*
